# Comparative In Vitro Activities of First and Second-Generation Ceragenins Alone and in Combination with Antibiotics Against Multidrug-Resistant *Klebsiella pneumoniae* Strains

**DOI:** 10.3390/antibiotics8030130

**Published:** 2019-08-27

**Authors:** Berna Ozbek-Celik, Damla Damar-Celik, Emel Mataraci-Kara, Cagla Bozkurt-Guzel, Paul B. Savage

**Affiliations:** 1 Department of Pharmaceutical Microbiology, Faculty of Pharmacy, Istanbul University, 34116 Beyazit-Istanbul, Turkey; 2 Department of Chemistry and Biochemistry, Brigham Young University, Provo, UT 84602, USA

**Keywords:** novel ceragenins, antibiotic, resistance, combination, gram negatives

## Abstract

Objectives: The ceragenins, or CSAs, were designed to mimic the activities of antimicrobial peptides and represent a new class of antimicrobial agent. The aim of this study was to comparatively investigate the antimicrobial activities of first/second generation ceragenins and various antibiotics against multidrug-resistant (MDR) *Klebsiella pneumoniae*, including colistin-resistant bacteria. Also, the synergistic effects of two ceragenins with colistin or meropenem were investigated with six *K. pneumoniae* strains presenting different resistant patterns. Methods: Minimal inhibition concentrations (MICs) were determined by the microdilution method according to the CLSI. Antibiotic combination studies were evaluated by the time–kill curve method. Results: MIC_50_ and MIC_90_ values of tested ceragenins ranged from 8 to 32 mg/L and 16 to 128 mg/L. Overall, among the ceragenins tested, CSA-131 showed the lowest MIC_50_ and MIC_90_ values against all microorganisms. The MICs of the ceragenins were similar or better than tested antibiotics, except for colistin. Synergistic activities of CSA-131 in combination with colistin was found for strains both at 1× MIC and 4× MIC. No antagonism was observed with any combination. Conclusions: First-generation ceragenins CSA-13 and CSA-44 and second-generation ceragenins CSA-131, CSA-138 and CSA-142 have significant antimicrobial effects on MDR *K. pneumoniae*. Mechanisms allowing resistance to clinical comparator antibiotics like colistin did not impact the activity of ceragenins. These results suggest that ceragenins may play a role in treating infections that are resistant to known antibiotics.

## 1. Introduction:

*Klebsiella pneumoniae* is highly prevalent in community-acquired and nosocomial infections [1]. The emergence and spread of multidrug-resistant *K. pneumoniae* may lead to a major therapeutic challenge, life-threatening infections, which are an important threat to global health with mortality rates of 40–50% [2]. These bacteria have become sequentially resistant to several classes of antibiotics including carbapenems, which are often the last resort for the treatment of infections due to extended-spectrum beta-lactamase (ESBL)-producing isolates worldwide [3]. A limited number of antimicrobial agents maintain effectiveness against carbapenem-resistant *K. pneumoniae*, including colistin and tigecycline. Considering the mortality rate associated with *K. pneumoniae* infections [4,5] and the prevalence of drug resistance, development of novel antimicrobials for these pathogens is critical. 

**Figure 1:**
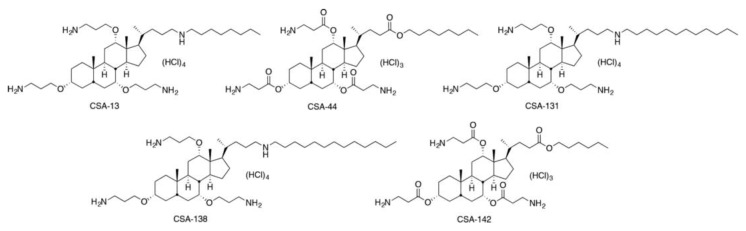
Structure of ceragenins used in this study.

Antimicrobial peptides (AMPs), such as human cathelicidin LL-37 and the defensins, play a central role in innate immunity. The importance of the activities of molecules becomes clear upon observation that AMPs are found in organisms ranging from insects to mammals; furthermore, most clinically relevant bacteria remain susceptible to AMPs. Recognition of these facts has prompted interest in development of AMPs for clinical use in preventing and treating bacterial infections [6,7,8]. However, clinical use of peptide therapeutics poses several problems: compounds of the complexity found among endogenous AMPs (ca. 20−50 amino acids) are relatively expensive to prepare, the activity of many AMPs is salt-sensitive and peptides are susceptible to proteolytic degradation [8]. Consequently, development of nonpeptide mimics of AMPs may provide a means of using the antimicrobial mechanisms evolved over years without the disadvantages of peptide therapeutics. Ceragenins are a family of bile acid derivatives that have been modified to yield an amphiphilic morphology similar to that of endogenous AMPs [9,10]. The majority of AMPs adopt amphiphilic secondary structures in which cationic amino acid side chains (i.e., arginine, lysine and histidine) are oriented on one face of the molecule while hydrophilic side chains are on the opposing face (Figure 1). In a manner similar to AMPs, ceragenins exhibit rapid bactericidal activity against a broad range of bacterial species [11,12]. However, the ceragenins have several advantages over AMPs, including their resistance to proteolysis and their amenability to large-scale synthesis. While most ceragenins are effective against both gram-negative and gram-positive bacteria, minimal inhibition concentrations (MICs) of ceragenins are typically lower against gram-positive organisms. The activities of these agents are exemplified by CSA-13, the most studied of the ceragenins with broad-spectrum antimicrobial activity. Additionally, previous studies showed that CSA-13 toxicity is comparable to LL-37 in tested human keratinocytes, and it is not toxic to HatCat cells at bactericidal concentrations [13].

The antibacterial activities of new ceragenins alone or in combination with other antibiotics have not been well characterized. In vitro studies of antibacterial activity may be used to select specific compounds for further development [14]. In the studies described herein, the in vitro activities of first-generation ceragenins (CSA-13, CSA-44), second-generation CSAs (CSA-131, CSA-138, CSA-142) against 50 strains of multidrug-resistant (MDR) *K. pneumoniae* were determined and compared to MICs of ceftazidime, colistin, tobramycin, levofloxacin and meropenem. In addition, activities of these ceragenins with colistin or meropenem were measured. The prevalence of infections caused by *K. pneumoniae* and the continuous emergence of multidrug-resistant pathogens highlight the need for development of new therapeutics, especially those that are unlikely to engender bacterial resistance.

## 2. Materials and Methods

### 2.1. Bacterial Isolates

For this study, 50 non-duplicate, nosocomially-acquired MDR *K. pneumoniae* isolates were collected from the Department of Infectious Diseases and Clinical Microbiology at the teaching hospital of Istanbul Medipol University (Istanbul, Turkey) in the first six months of 2017. These 50 strains were isolated from the following sources; 15 from the respiratory tract, 26 from blood, 9 from sputum. All strains were identified using API 20E (bioMérieux, Marcy-l’Étoile, France). As a reference strain, *Escherichia coli* ATCC 25922 (American Type Culture Collection, Rockville, MD, USA) was used throughout the susceptibility studies.

### 2.2. Antimicrobial Agents

Antibioticswere kindly provided by their respective manufacturers. Stock solutions of ceftazidime (GlaxoSmithKline, Istanbul Turkey), colistin sulphate (Sigma-Aldrich, St. Louis, MO, USA), levofloxacin (Sanofi Pharmaceuticals Inc, Istanbul, Turkey) and tobramycin (Novartis Pharmaceuticals Corp., Istanbul, Turkey) were prepared from dry powders at a concentration of 5120 mg/L and were stored frozen at −80 °C. Frozen solutions of antibiotics were used within 6 months. Meropenem solutions were prepared on the day of use. Ceragenins (Figure 1) were provided by one of the authors (P.B.S.) and were synthesized from a cholic acid scaffolding technique as previously described [15].

### 2.3. Media

Freshly prepared Mueller–Hinton broth (Oxoid Ltd., Basingstoke, UK) was supplemented with 25 mg/L calcium and 12.5 mg/L magnesium and was used for MIC determination and combination studies. Tryptic soy agar (Oxoid Ltd.) was used for time–kill assays.

### 2.4. MICs 

MICs were determined by the microbroth dilution technique according to the Clinical and Laboratory Standards Institute (CLSI) [16]. Serial two-fold dilutions ranging from 256 to 0.125 mg/L for ceftazidime, from 128 to 0.06 mg/L for meropenem, levofloxacin and CSAs, and from 32 to 0.015 mg/L for tobramycin and colistin were prepared in cation-supplemented Mueller–Hinton broth in 96-well microtiter plates (TPP, Trasadingen, Switzerland).

### 2.5. Time–Kill Studies

Time–kill assays were performed on six isolates representing different susceptibility patterns (two of them were colistin-resistant/meropenem-susceptible MDR strains and two of them were MDR colistin-susceptible/meropenem-resistant strains and the last two were colistin and meropenem-susceptible MDR strains). To evaluate concentration-dependent bactericidal activity, these strains were exposed to one first-generation ceragenin, CSA-13, one second-generation ceragenin, CSA-131, meropenem and colistin alone at 1× and 4× MIC. The reason for the selection of these agents is that they were identified as the most effective antimicrobials from our MIC experiments. Also, CSA-13 in combination with colistin or meropenem and CSA-131 in combination with colistin or meropenem were tested at 1× and 4× MIC following the methods published by the National Committee for Clinical Laboratory Standards (NCCLS) [17]. Time–kill assays were sampled for colony counts at 0, 2, 4, 6 and 24 h. The lower limit of detection by this method was 20 CFU/mL. According to the NCCLS criteria, synergy and antagonism were defined as a ≥2 log10 decrease or increase, respectively, in CFU/mL at 24 h for antibiotic combinations as compared with its more active constituent. Indifference was defined as a <2 log10 change (increase or decrease) in colony count at 24 h by the combination in comparison with the most active single antimicrobial alone. Bactericidal activity was defined as ≥3 log10 CFU/mL reduction compared with the initial inoculums within 24 h.

## 3. Results

The in vitro activities of the antibiotics studied against 50 clinical isolates of MDR *K. pneumoniae* are summarized in Table 1. All strains were selected for decreased susceptibility to at least three different antibiotics groups and according to CLSI breakpoints, they were all MDR strains. All strains were resistant to ceftazidime (MIC_90_, 512 mg/L), 13 strains were resistant to colistin (MIC_90_, 64 mg/L), 46 strains were resistant to meropenem (MIC_90_, 128 mg/L), 47 strains were resistant to levofloxacin (MIC_90_, 64 mg/L), and to tobramycin (MIC_90_, 256 mg/L). Based on MIC_90_ values, colistin was the most potent agent, followed by meropenem. 

Ceragenins CSA-13, CSA-44, CSA-131, CSA-138, CSA-142 were also investigated for their activity against MDR *K. pneumoniae* strains. Their MIC_90_’s were determined as 32 mg/L for CSA-13 and CSA-44, 16 mg/L for CSA-131, 32 mg/L for CSA-138 and 128 mg/L for CSA-142.

**Figure 2:**
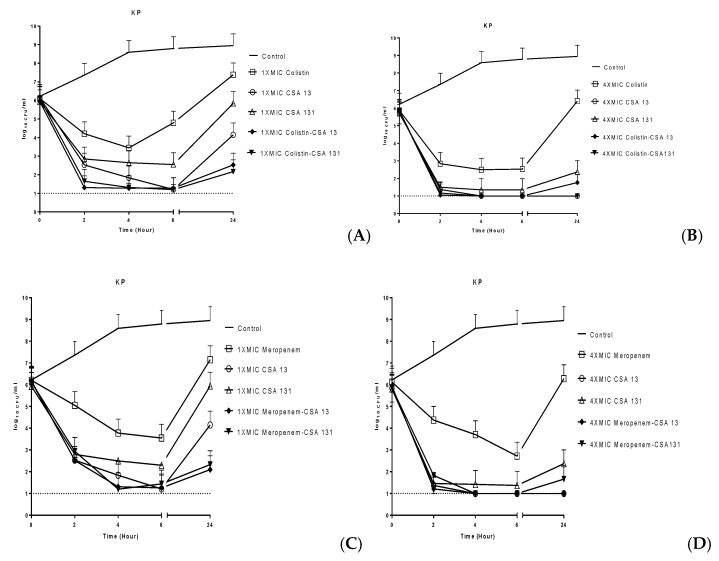
The mean results of killing activity of two CSAs with colistin or meropenem combinations against six *K. pneumoniae* strains.

Time–kill assays were performed on six isolates representing different susceptibility patterns and the results are given in Figure 2. According to the mean results of the time–kill curve studies, meropenem was found bactericidal (>3 log10 reduction in live cell count) only at 6 h at 4× MIC. On the other hand, colistin was found bactericidal at 2 h, 4 h and 6 h at 4× MIC. Although both CSA-13 and CSA-131 were found bactericidal at 2 h, 4 h and 6 h at the two concentrations tested, these ceragenins maintained their bactericidal effects at 24 h only at 4× MIC. With CSA-13 at 4× MIC, (≥5 log10 reduction in live cell count of all MDR-phenotype isolates was observed. 

To evaluate possible synergy, indifference or antagonism between antimicrobials used in pairs, standards outlined by the NCCIS/CLSI were used. Specifically, these are that synergy and antagonism are defined as a two-log decrease or increase, respectively, in CFU/mL at 24 h for antibiotic combinations as compared with the more active constituent alone. Indifference is defined as a less than two-log change (increase or decrease) in colony count at 24 h by the combination in comparison with the more active single antimicrobial alone. In combination, CSA-13 and colistin showed additive antibacterial activity for all six of the strains, while the combination of CSA-131 and colistin resulted in synergistic activity for three of the six strains at 1× MIC. Average bacterial counts from experiments with the six strains are given in Figure 2. Data for individual strains are included in the supporting information. The CSA-13 or CSA-131 with meropenem combinations were measured as additive against all six strains at both 1× MIC and 4× MIC. No antagonism was observed with any combination. 

## 4. Discussion

Although carbapenems have been used as monotherapy in the treatment of patients with *K. pneumoniae*, the emergence of carbapenem resistance in these bacteria has become a global concern. Even more disconcerting is the fact that many carbapenemase-producing strains are resistant to multiple other antibiotic classes [18,19,20]. Similar to previous studies describing resistance profiles of *K. pneumoniae* isolates [21,22], we found 26% of isolates studied were colistin-resistant, 88% of the strains were meropenem-resistant, 92% of the strains were tobramycin-resistant, 96% of the strains were levofloxacin-resistant and all of the strains were ceftazidime-resistant. When the activities of single antibiotic agents were compared, colistin was inferior to meropenem, tobramycin or levofloxacin and ceftazidime against these MDR strains. Although colistin, a polymyxin antibiotic, is used in the treatment of MDR, especially *K. pneumoniae*, this high resistance rate of colistin might reflect the extensive use of antibiotics in clinics in Turkey, and it shows that colistin monotherapy should only be used after a susceptibility test has been performed. 

First- and second-generation ceragenins are new molecules which display antimicrobial activity against a wide spectrum of bacteria [23,24,25,26]. Chin et al. evaluated activity of CSA-13 against clinical isolates of *Pseudomonas aeruginosa*, and the MIC_50_ of the 50 clinical isolates in that study was found to be 16 mg/L [10]. A previous study showed that ceragenins are active against colistin-resistant clinical isolates of *K. pneumoniae* [27]; Hashemi et al. showed that colistin-resistant clinical isolates of *K. pneumoniae* gave MICs of 16 to 200 mg/L with colistin, while with the ceragenins their MICs were relatively low; 2–6 mg/L for CSA-13, 1–2 mg/L for CSA-44, 1–3 mg/L for CSA-131, 1–8 mg/L for CSA-138 and 2–16 mg/L for CSA-142. In the present study, all ceragenins, except CSA-142, gave at least two-fold lower MICs, against 50 and 90% (MIC_50_ and MIC_90_) of strains tested, than tobramycin, levofloxacin and ceftazidime, and they were active against all isolates at concentrations comparable to meropenem. The MIC_50_s of the all tested ceragenins were four- to eight-fold higher than colistin, while the MIC_90_s were two- to four-fold lower than those for colistin. According to their MIC_90_ values, the activity of the five ceragenins ranks as follows: CSA-131 < CSA-13, CSA-44, CSA-138 < CSA-148. CSA-131 showed the best activity against the MDR strains tested, with MIC_50_ of 8 mg/L and MIC_90_ 16 mg/L. 

In a previous study, the antibacterial activity of ceragenins against gram-negative bacteria was shown to be dependent in part on the length of the lipid chain extending from the molecule [28]. It was proposed that this lipid chain provides an anchor into the outer membrane of the bacteria. In the studies presented herein, CSA-131, with a 12-carbon lipid chain, showed better activity than CSA-13 and CSA-44, both with eight-carbon lipid chains (Table 1). Ceragenin CSA-142, with a six-carbon chain was the least active. Interestingly, the length of the carbon chain cannot be extended indefinitely to achieve more active compounds; ceragenins with chain lengths beyond 12 begin to lose activity, and the addition of one carbon to CSA-131, to give CSA-138, results in a loss of activity.

To quantify rates of reduction in bacterial inocula, population reduction assays were performed with CSA-13 and CSA-131 against six strains and compared to colistin and meropenem (Figure 2). At 4× MIC for both ceragenins, the inoculum was decreased by at least three logs within 24 h. Also at 4× MIC, the inocula were decreased to the detection limit (one log) within the same time frame for CSA-13. It should be noted that CSA-13 and CSA-131 substantially reduced bacterial populations against both meropenem- and colistin-resistant *K. pneumoniae* strains at 1× MIC and 4× MIC. Similar to results from the previous study by Pollard et al. [29], we found that there are only minor differences in the kinetics of antimicrobial activity of ceragenins among the colistin-resistant and colistin-susceptible strains or the meropenem-resistant and meropenem-susceptible strains. These results suggest that colistin resistance and meropenem resistance do not significantly influence susceptibility to ceragenins. Additionally, CSA-13 and CSA-131 gave substantial decreases in bacterial counts (greater than three-log reductions) at all of the tested concentrations. Notably, bacterial re-growth did not occur for CSA-13 and CSA-131 at 24 h against both resistant and susceptible strains.

The abilities of these ceragenins to hold bacterial populations in check may allow the endogenous antimicrobial activities of the innate immune system of higher organisms to continue to suppress bacterial growth. Synergy between innate immune function and ceragenins may allow effective use of concentrations of ceragenins below 1× MIC. Synergy between ceragenins and other antibiotics has been described [30,31], and it has been proposed that synergism comes from interactions of ceragenins with lipid A in the outer membranes of gram-negative bacteria that reduce the permeability barrier provided by the membranes [32]. The synergy observed with CSA-131 and colistin is also likely due to enhanced transport of colistin across the outer membranes. 

## 5. Conclusions

Overall, the MICs of ceragenins against colistin- and meropenem-resistant strains and their high antibacterial activity over 24 h make these compounds attractive investigational therapeutics for treating infections caused by drug-resistant bacteria.

## Figures and Tables

**Table 1:** Comparative in vitro activity of antimicrobial agents against 50 isolates of multidrug-resistant (MDR) *K. pneumoniae* (mg/L).

Antibiotics	MIC range	MIC_50_	MIC_90_	Percent inhibited at CLSI breakpoints ^a^	
				S	R
Ceftazidime	16->512	256	>512	0	100
Colistin	0.03–128	0.25	64	74	26
Meropenem	0.5–>128	32	128	8	92
Levofloxacin	0.25–>128	32	64	6	94
					
Tobramycin	4–512	64	256	12	88
CSA-13	0.5–32	16	32	-	-
CSA-44	0.5–32	16	32	-	-
CSA-131	0.5–16	8	16	-	-
CSA-138	1–32	16	32	-	-
CSA-142	1–128	32	128	-	-

-, No data. MIC, minimal inhibition concentrations. ^a^ CLSI breakpoints for *Enterobacteriaceae* for susceptibility and resistance to ceftazidime are ≤8 mg/L and ≥32 mg/L, for colistin are ≤2 mg/L and ≥4 mg/L, for meropenem are ≤4 mg/L and ≥16 mg/L, for levofloxacin are ≤2 mg/L and ≥8mg/L, for tobramycin are ≤4 mg/L and ≥16 mg/L. S; susceptible, R; resistant
